# The Role of MicroRNA Signature as Diagnostic Biomarkers in Different Clinical Stages of Colorectal Cancer

**DOI:** 10.22074/cellj.2018.5366

**Published:** 2018-03-18

**Authors:** Sara Eslamizadeh, Mansour Heidari, Shahram Agah, Ebrahim Faghihloo, Hossein Ghazi, Alireza Mirzaei, Abolfazl Akbari

**Affiliations:** 1 Department of Molecular Genetics, Marvdasht Branch, Islamic Azad University, Marvdasht, Iran; 2 Department of Molecular Genetics, Science and Research Branch, Islamic Azad University, Fars, Iran; 3 Department of Molecular Biology and Genetics, Bushehr Branch, Islamic Azad University, Bushehr, Iran; 4 Colorectal Research Center, Iran University of Medical Sciences, Tehran, Iran; 5 Department of Microbiology, School of Medicine, Shahid Beheshti University of Medical Sciences, Tehran, Iran; 6 Ophthalmic Research Center, Shahid Beheshti University of Medical Sciences, Tehran, Iran; 7 Bone and Joint Reconstruction Research Center, Shafa Orthopedic Hospital, Iran University of Medical Sciences, Tehran, Iran

**Keywords:** Biomarker, Blood, Colorectal Cancer, Diagnosis, MicroRNA

## Abstract

**Objective:**

Colorectal cancer (CRC) is one of the most common cancers and a major cause of cancer-related death worldwide. The early diagnosis of colorectal tumors is one of the most important challenges in cancer management. MicroRNAs (miRNAs) have provided new insight into CRC development and have been suggested as reliable and stable biomarkers for diagnosis and prognosis. The aim of this study was to analyze the differential expression of miRNAs at different stages of CRC searching for possible correlation with clinicopathological features to examine their potential value as diagnostic biomarkers.

**Materials and Methods:**

In this case-control study, plasma and matched tissue samples were collected from 74 CRC patients at stage II-IV as well as blood samples from 32 healthy controls. After exhaustive study of the current literature, eight miRNAs including miR-200c, 20a, 21, 31,135b, 133b,145 and let-7g were selected. The expression level of the miRNAs was assayed by quantitative reverse transcriptase-polymerase chain reaction (qRT-PCR). Statistical analysis, including t test , Mann-Whitney U, Kruskall-Wallis tests and receiver operating characteristic (ROC) curve was applied, where needed.

**Results:**

Significantly elevated levels of miR-21, miR-31, miR-20a, miR-135b, and decreased levels of miR- 200c, miR-145 and let-7 g were detected in both plasma and matched tissue samples compared to the healthy group (P<0.05). However, no significant differences were observed in the expression level of plasma and tissue miR-133b (P>0.05). ROC for tissue miRNAs showed an area under the ROC curve (AUC) of 0.98 and P<0.001 for miR-21, 0.91 and P<0.001 for miR-135b, 0.91 and P<0.001 for miR-31, and 0.92 and P<0.001 for miR-20a.

**Conclusion:**

Our results indicate that the expression levels of microRNAs are systematically altered in CRC tissue and plasma. In conclusion, detection of miR-21, miR-135b, miR-31 and miR-20a levels in the tissue might be helpful to illuminate the molecular mechanisms underlying CRC carcinogenesis and serve as tumor-associated biomarkers for diagnosis.

## Introduction

Colorectal cancer (CRC) is one of the most commonly diagnosed types of cancer in men and women, worldwide. Each year, more than 1 million new cancer- related cases are diagnosed, causing about 600,000 deaths (1). Among Asian populations, both the incidence and mortality rates of CRC are increasing rapidly (2, 3). CRC is a complex disease, charactrized by anumber of genetic alterations and dysregulated signaling pathways (1). Since this malignancy is mainly asymptomatic and often diagnosed at the late stage with poor prognosis, a great emphasis is placed on early tumor detection to decrease the mortality rate (4). 

Colonoscopy is currently the gold standard and has very high sensitivity and specificity in detecting colorectal lesions, but because of its invasive nature and other limitations, is not considered as the best method of choice. Therefore, there is an urgent need for identifying stable, specific and reliable biomarkers to facilitate the early detection of CRC (5). 

MicroRNAs (miRNAs), as well-known non-coding RNAs, have been demonstrated to be involvedin cancer development and progression. The major mechanisms of actions of most miRNAs involves translational inhibition and mRNA degradation, which is mediated by complementary binding to target mRNAs (6, 7). Studies have indicated that miRNAs can control hundreds of target genes via their oncogenic or tumor- suppressive activities depend on the target (6).

Since their discovery, microRNAs have been found to regulate cellular processes which have key roles in different aspects of cancer biology, such as cell proliferation, apoptosis and differentiation (6, 7). Therefore, these molecules have recently achieved remarkable success in cancer management through their regulatory roles in gene expression and protein translation (8). There is increasing evidences that miRNAs are largely dysregulated in different types of solid cancerous tissues and their tissue-specific expression profiles have promising applications in cancer diagnosis, prognosis and treatment (9).

Based on this background, identification of the altered miRNAs expression profiles as well as their association with tumorigenesis are important steps in cancer management (9, 10). On the other hand, it has been distinguished that the altered expression of miRNAs in a range of malignant tissues could be reflected in circulation, suggesting them as promising diagnostic biomarkers (10-12). A number of studies have also confirmed the potential value of circulating miRNAs as diagnostic and prognostic biomarkers for CRC (11, 12). However, to gain an insight into the role of miRNAs in CRC carcinogenesis, identifying their association with clinicopathological features is especially helpful (13).

In this study, eight miRNAs (miR-21, miR-135b, miR-133, miR-let7-g, miR-31, miR-20a, miR-200c and miR-145) were particularly chosen based on comprehensive review of the literature and other previously published data on CRC. MiR-21, miR135b, miR-20a and miR-31 have been suggested as oncogenic regulators in CRC, whilst, let7-g, miR-145 and miR-133b act as tumor suppressors.

These miRNAs were selected for their potentials as diagnostic biomarkers and probable biological significance in CRC (10, 14, 15). However, it has been reported that miR-200c could act as an oncogene or tumor suppressor in CRC depending on TNM stage (13, 15-17). The purpose of this study was to examine the differential expression of CRC-associated miRNAs in tissue and plasma from different stages of the disease to evaluate their clinical value as diagnostic biomarkers.

## Materials and Methods

In this case-control study, a total of 74 blood samples (39 with stage II, 30 with stage III and 5 with stage IV of CRC) and a subset of 74 matched tumor tissues were collected from CRC patients. Histopathological features were confirmed by pathological analysis and the patients were staged according to the tumor-node metastasis (TNM) staging system of the International Union Against Cancer (16).

This study has been approved in Ethic Committee of Iran University of Medical Sciences (Ethical code: IR.IUMS. REC 1394.26649). Written and signed informed consent forms were gathered from all the volunteers participating in this study. Participation of individuals was voluntary and all participants were aware of the project’s purpose. The patients had received no chemotherapy. Also, all participants stated they had received no treatment in the two months prior to the study. In the healthy group, 32 blood samples were collected from subjects with no current malignancy or infectious disease. The healthy subjects were matched to the cancer patients, according to age and gender.

### Sample collection and preparation

The surgical tumor samples were microscopically inspected by an oncologist, who obtained a biopsy from a section representative of the colon or rectum tumor. The tumor tissue specimens were placed directly in RNALater (Thermo Fisher scientific, Germany) and transferred to the molecular laboratory and stored at -20°C until use. Peripheral blood was collected in vacutainer liquid EDTA 6 ml blood collection tubes, and peripheral blood mononuclear cells (PBMCs) Cs and plasma fractions were separated by density gradient separation. Then, the upper layer (plasma) was stored in 2 ml DNase/RNase free microtubes and frozen at -80°C until miRNA extraction.

### miRNA extraction

The plasma (200 µl) and tumor tissue (5 mg) samples were subjected to miRNA extraction. Frozen plasma samples were thawed and miRNAs were extracted using a miRNA extraction kit (Qiagen, Valencia, CA, USA) according to the manufacturer’s instructions. Tissue miRNAs (tumor and the corresponding normal tissues) were isolated by a modified TRIzol protocol as explained previously (14). The quantity and quality of the extracted RNA was evaluated using spectrophotometry and gel electrophoresis, respectively. 

### cDNA synthesis

Total RNA (1 µg) was reverse transcribed into cDNA using cDNA Reverse Transcription Kit (Ampliqon, Denmark) as explained previously (16, 17). Briefly, 1 µg of RNA was mixed with 1 µl of random hexamer primers and 1 µl M-MuLVreverse transcriptase (200 U/ µl). Nuclease-free diethyl pyrocarbonate (DEPC)-treated water was added to bring the mixture up to a volume of 15 µl. Then, the mixture was incubated at 65°C for 5 minutes in a 7500 thermocycler (ABI) and cDNA was synthesized with the program of 5 minutes at 25°C, 60 minutes at 42°C, and 5 minutes at 70°C.

### Bioinformatics study and miRNA selection

Eight miRNAs selected by virtue of being demonstrated as colorectal cancer-associated miRNAs and established as a tumor suppressors or an oncogenes in CRC. The miRNAs involved in colon cancer, were selected from the Sanger Center miRNA Registry at http://www.sanger.ac.uk/Software/ Rfam/mirna/index.shtml. Furthermore, we used some miRNA databases (miRDB, TargetScan, miRBase and miRTarBase) to predict the biological targets of miRNAs and validate microRNA-target interactions both *in vitro* and *in vivo*.

### Quantitative reverse transcriptase polymerase chain reaction for detecting mature miRNAs 

The expression of a for ementioned miRNAs was measured by a poly A quantitative reverse transcriptase polymerase chain reaction (qRT-PCR) technique using specific oligonucleotide primers (14). All PCR reactions for CRC and control samples were performed in duplicate and the mean CT data was obtained using cycle threshold settings. The relative expression levels of miRNAs in tissue and plasma were normalized to that of RNU6B asaninternal control. The normalization was completed by using the equation: log_10_ (2^-ΔΔCt^), in which ΔΔCt =Ct_CRC^-^_ Ct_control_(18).

### Statistical analysis 

At first, the normality of the data was assessed using the Kolmogorov-Smirnov test. Data were analyzed using the independent t test, when the distribution was normal, and Mann-Whitney U test, when the distribution was not normal. To evaluate the diagnostic value of the miRNAs, receiver operating characteristics (ROC) curve was completed. Level of significance for statistical tests was 0.05. The SPSS software version 22 was used for the analyses.

## Results

### Clinicopathological characteristics of the studied colorectal cancer patients 

Clinicopathological features of the CRC patients have been detailed. There was no evidence of further disease complications in the participants. Conerning location of the tumors, 47 (63.5%) patients had rectum tumors, and 27 (36.5%) located in the colon. Moreover, 39 participants (52.7%) had stage II, 30 (40.5%) had stage III, while 5 (6.7%) had a stage IV CRC. The patients included 48 males (64.86%) and 26 females (35.13%) with ages rangingfrom 29 to 84 years old (median age: 49.6 years). The clinical and pathological characteristics have been described (Table 1).

### Plasma miRNAs levels

The results demonstrated that all studied miRNAs (miR200c, miR-145, miR-135b, miR-133b, miR-31, miR-21, and miR-20a and let-7 g) were differentially expressed either in plasma or tissue samples from CRC patients compared to healthy controls. Based on the Kolmogorov-Smirnov test, these miRNA values were not normally distributed (P<0.05). Therefore, the Mann-Whitney U test was used to compare the expression of miRNAs between the CRC patients and controls. However, independent t test was applied to compare the expression of miRNAs between the CRC patients and controls. qPCR data indicated that the expression levels of plasma miR-20a, miR-21, miR31, miR-135b were significantly higher than those in the healthy controls (P<0.05, Figes. 1, 2). By contrast, the expression levels of miR-145, miR-let-7g and miR-200c were significantly lower (P<0.05) in the plasma of CRC patients than that of the healthy controls (P<0.05, Fig .3).

**Table 1:** Clinicopathological features of the studied CRC patients


Variable		Number of RNA samples n=74

Age (Y)		
	≥55	33
	<55	41
Gender		
	Male	48
	Female	26
TNM stage		
	II	39
	III	30
	IV	5
Tumor size		
	<2 cm	10
	2-3.5 cm	31
	3.5-5 cm	24
	>5 cm	9
Localization		
Colon	27
Rectum	47
LVI		
	Positive	46
	Negative	28
Differentiation		
	Well Adeno	11
	Moderate Adeno	59
	Poor Adeno	4


CRC; Colorectal cancer, TNM; Tumor-node-metastasis, and LVI; Lympho vascular invasion.

### Expression of miRNAs in colon cancer tissues

Based on the qPCR data, a statistically significant (P<0.001) up-regulation of miR-20a, miR-21, miR-31 and miR-135b was found in the CRC tissues compared to normal adjacent mucosa. Conversely, the expression levels of miR-200c, miR-145 and let-7g were signific antly lower in tumor tissue compared to adjacent normal tissues (P<0.001, Figes.2, 4). However, no significant differences were found either in the expression level of plasma or tissue miR-133b between CRC patients and healthy controls (P>0.05).

**Fig.1:**
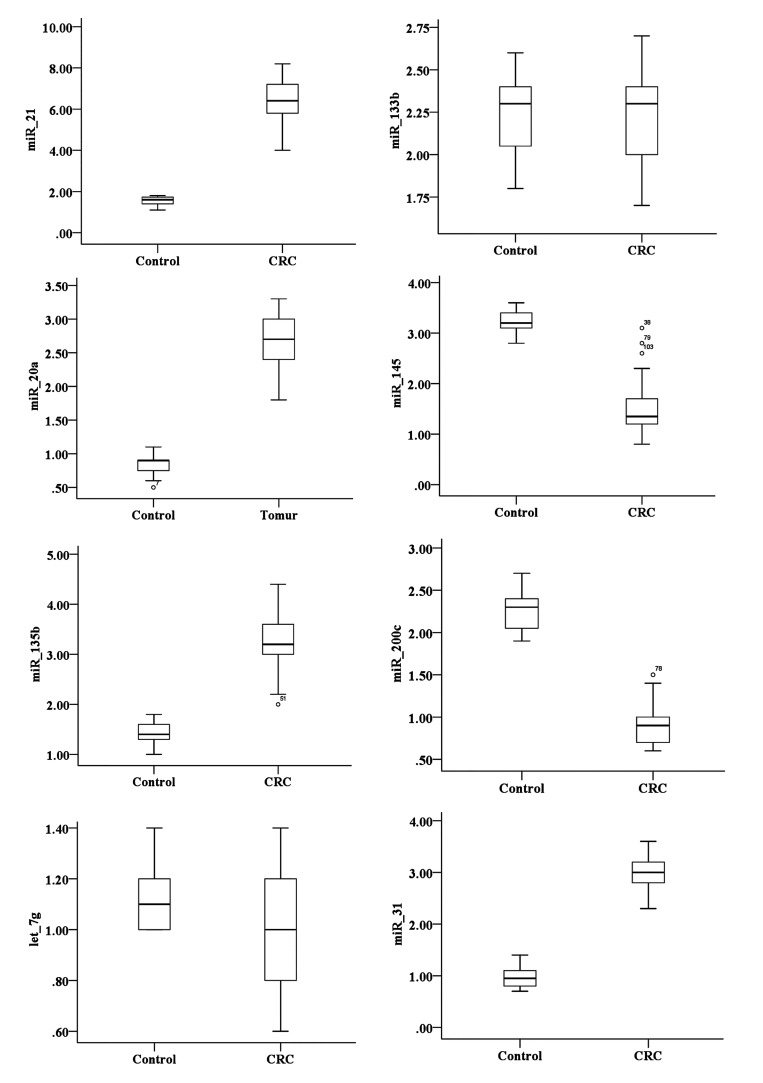
The relative expression of miR-31, miR-200c, miR-20a, miR-135b, miR-133b, miR-21, miR-145 and let-7g in tissue [74 colorectal cancer (CRC) samples compared to 32 controls]. Lines in the middle show the mean expression value.

**Fig.2:**
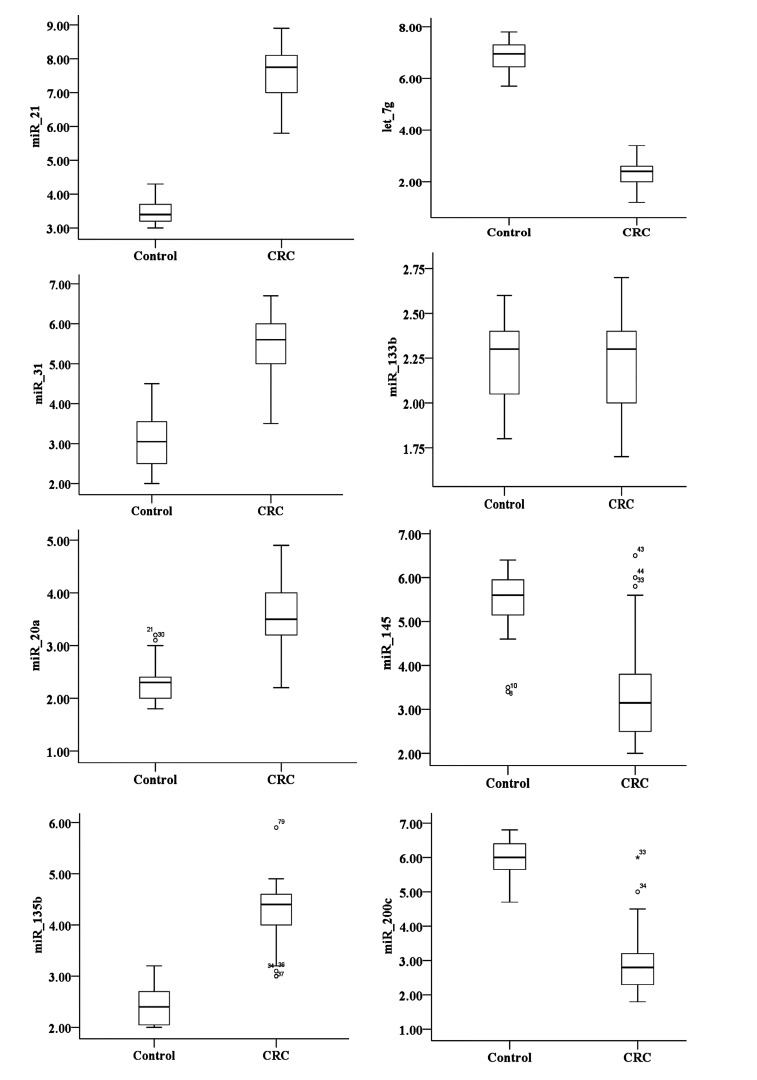
The relative expression of miR-31, miR-200c, miR-20a, miR-135b, miR-133b, miR-21, miR-145 and let-7g in plasma [74 colorectal cancer (CRC) samples compared to 32 controls]. Lines in the middle show the mean expression value.

**Fig.3:**
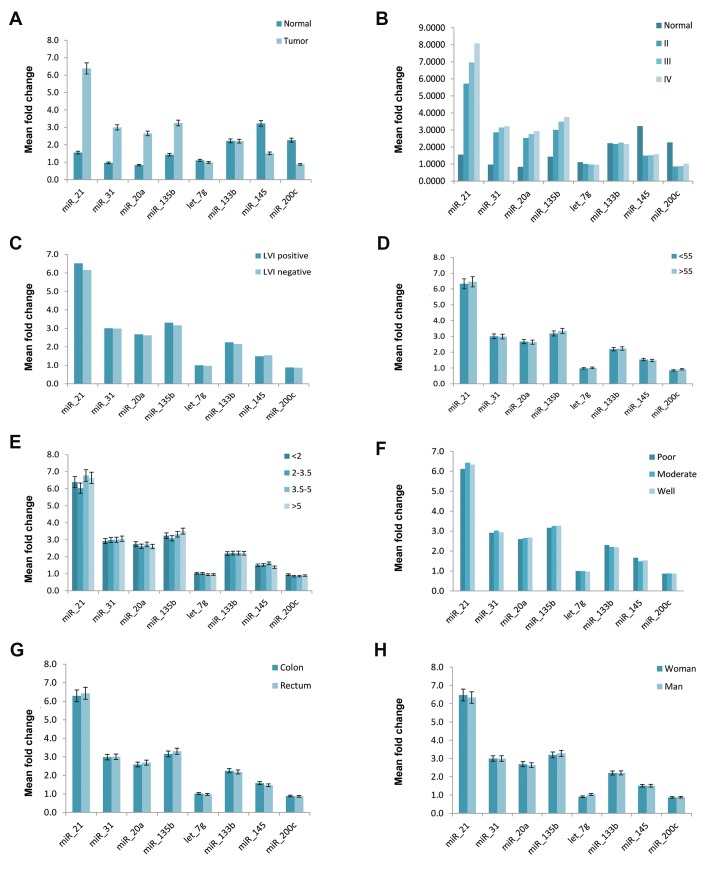
Correlation of clinicopathologic features of colorectal cancer with the relative expression levels of miRNAs in plasma. A. Comparison of miRNA levels in colorectal cancer (CRC) and healthy controls, B. Comparison of miRNA expression level in patients with different tumor stages, C. Comparison of miRNA expression levels in different lymphovascular invasion (LVI) status, D. Comparison of miRNA expression levels in CRC according to age, E. Comparison of miRNA levels in CRC with different tumor sizes, F. Comparison of miRNA expression levels in colon and rectal cancers, G. Comparison of miRNA expression levels in patients with various tumor differentiation, and H. Comparison of miRNA expression levels in CRC based on patient’s gender.

**Fig.4:**
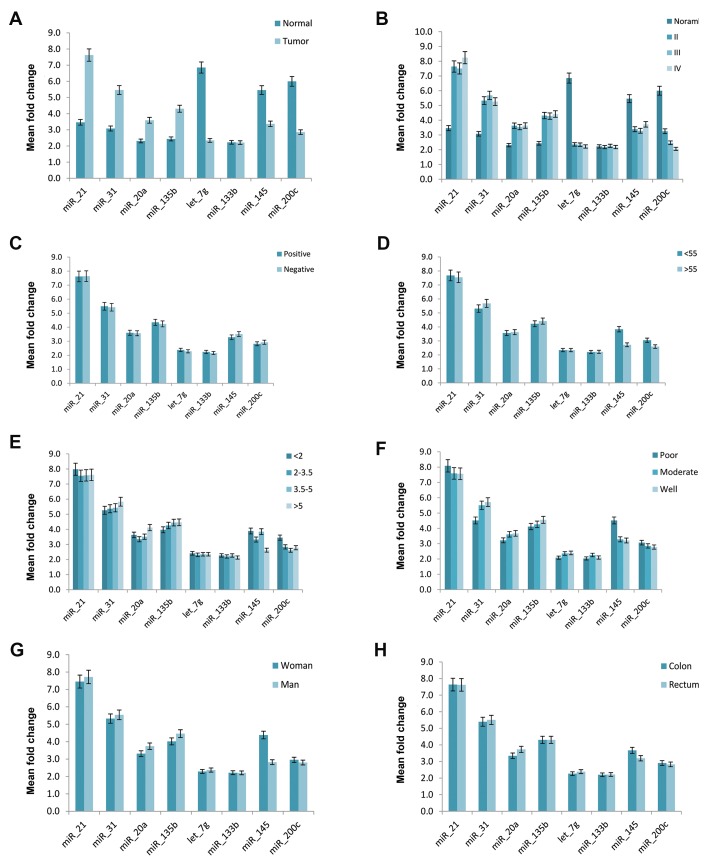
Correlation of clinicopathological features of colorectal cancer (CRC) with the tissue expression level of miRNAs. A. Comparison of miRNA expression levels in CRC and healthy controls, B. Comparison of miRNA expression levels in patients with various tumor stages, C. Comparison of miRNA expression levels in CRC in different lymphovascular invasion (LVI) status, D. Comparison of miRNA expression level in CRC according to patient’s age, E. Comparisonof miRNA expression level in CRC with different tumorsizes, F. Comparison of miRNA expression levels in colon and rectal cancers, G. Comparison of miRNA expression levels in patients with different tumor differentiation, and H. Comparison of miRNA expressionlevels in CRC based on patient’s gender.

### The correlation of miRNA expression between matched tissue and plasma samples

**Fig.5:**
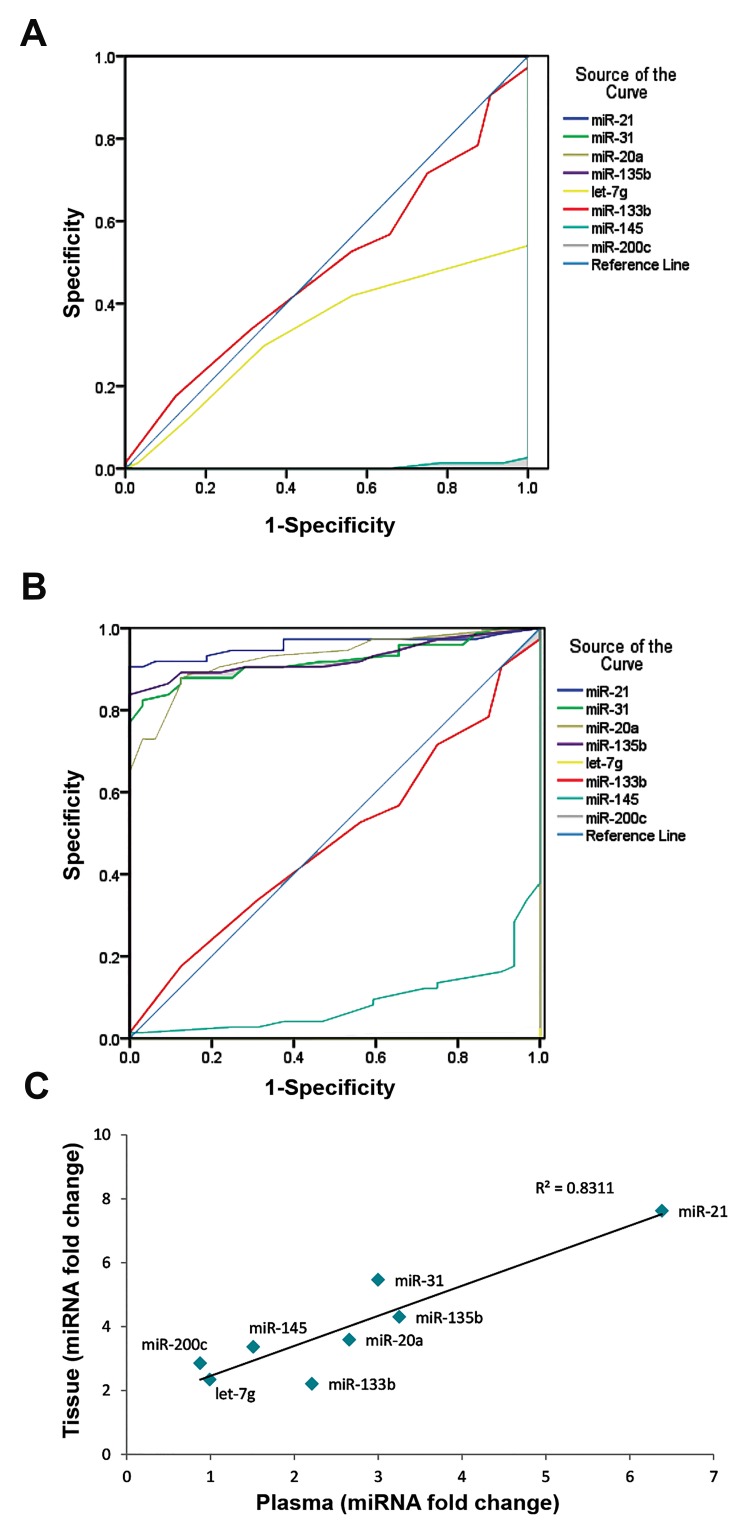
Evaluation of diagnostic power of miRNAs. A. Receiver operating characteristic (ROC) curve analysis using plasma, B. Tissue miRNAs expressionlevels for distinguishing colorectal cancer (CRC) samples (74 cancer samplesand 32 healthy controls), and C. Pearson correlation scatter plot of miRNAexpression levels in colorectal cancer tissue and plasma.

Investigation of miRNA expression levels in tissue and matched plasma samples showed that the relative trends of miRNA expression are similar in both tissue and plasma samples. The correlation analysis of expression of selected miRNAs expression showed aimportantcorrelation of miRNA expression patterns in the tissues with those in the plasma, R^2^=0.831 (Fig .5). It suggested that plasma miRNA patterns accurately reflect the expression signature of their tissue counterparts. 

### Clinicopathologic features of colorectal cancer and miRNA expression

Further analysis wasperformed to examine whether there was an association between miRNA expression levels and different clinicopathologic features including tumor stages, age, gender, tumor size, differentiation and lymphovascular invasion (LVI) status. Characteristic stage-dependent variation in the expression level of tissue and plasma miRNAs were analyzed between various stages (II, III and IV) of CRC. The results showed miR21, miR-31, miR-20a, miR-135b were significantly upregulated in CRC (tumor stages II, III, IV) compared to normal colorectal tissues (P<0.05). In contrast, miR-145, let-7g and miR-200c were significantly downregulated in CRC (tumor stages II, III, IV) compared to normal tissues (P<0.05). 

The plasma levels of miR-21, miR-31, miR-20a and miR135b showed a significant rising with the higherstages of malignancy (P<0.05). By contrast, miR-145, miR-let-7g and miR-200c showed a significant decreasing trend with the higher stages of malignancy (P<0.05). Additionally, the plasma levels of let-7g showed a significant decrease in stage III compared to healthy control (P<0.05).

The expression levels of miR-21, miR-31and miR135b in CRC plasma samples were significantly different between patients with stage II and III (P<0.05). Further analysis revealed no remarkable correlations between plasma and tissue levels of miRNAs and other clinicopathological features such as tumor size, tumor differentiation and LVI status (Figes.3, 4).

### Evaluation of the diagnostic power of miRNAs

To confirm the diagnostic value of the miRNAs signature, ROC curve analysis was performed for both the plasma and tissue data. The test demonstrated significant accuracy in discriminating CRC patients from healthy individuals for tissue miR-135b, miR-31, miR-21 and miR-20a. The calculating ROC AUC for tissue miRNAs was 0.98 and P<0.001 for miR-21, 0.91 and P<0.001 for miR-135b, 0.91 and P<0.001 for miR-31, and 0.92 and P<0.001 for miR-20a, which was suggestive of high discriminatory power (Fig .5).

## Discussion

CRC is one of the deadliest malignancies that is frequently diagnosed at advanced stages with poor prognosis (1, 2). Therefore, there is an urgent need for finding stable and reliable biomarkers to facilitate early detection of the disease and decrease mortality rates (5, 12). Micro RNAs (miRNAs), as a well-known group of non-coding RNAs, have been revealed to play a vital role in CRC carcinogenesis (7) . In addition, it has been suggested that CRC-associated miRNAs might serve as tissue-based biomarkers for cancer classification and diagnosis (6, 9). The circulating cell-free miRNAs have been regularly investigated in serum/plasma of cancer patients and reported to bestable biomarkers in various malignancies including, CRC (5, 11). Nevertheless, various nucleic acids released from blood cells into serum during coagulation and the right spectrum of tumor- derived circulating miRNAs fluctuates in serum samples. Since serum may potentially contain blood cell-derived miRNAs (15), we postulated the plasma samples can bemore reliable sources of tumor-derived circulating miRNAs. 

Considering the oncogenic or suppressor roles designated for different miRNAs in cancer development (6, 9), it is valuable to identify CRC-related miRNA signatures to expand our knowledge of their biological function. Here, we investigated the pattern of plasma miRNAlevels as well as matched tissue samples in CRC patients in comparison with healthy individuals and analyzed the potential value of these molecules as diagnostic biomarkers. In line with this aim, eight miRNAs (miR-21, miR-135b, miR-133, miR-let7-g, miR-31, miR-20a, miR-200c and miR-135b) were selected by virtue of having been demonstrated to be potential biomarkers for CRC (10, 13, 14).

The expression analysis demonstrated that all studied miRNAs were differentially expressed in patients compared to healthy controls. Our results indicated the same altered expression pattern in both plasma and tissue samples. We observed significantly elevated levels of miR-21, miR-31, miR-20a and miR-135b and significantly decreased levels of miR-145, miR-200c and miR-let-7g in both plasma and matched tissue samples compared to the healthy groups.

However, no significant differences were observed in the expression level of miR-133b either in plasma or tissue, between CRC patients and healthy controls. As well, the supplementary analysis demonstrated a significant correlation of miRNA expression levels in the CRC tissues and those in the plasma, with R^2^=0.831. These data illustrated that, the manner of miRNAs expression in plasma is similar to their corresponding tissues.

A forementioned findings suggested that the plasma miRNA expression pattern in CRC could reflect the expression signature of the tumor tissue. It seems that, the CRC tumor-derived miRNAs could be release into the blood stream. Consistent with this, it has been confirmed that the epithelial tumor-derived miRNAs potentially enter into and can be detected in circulation. Furthermore, we evaluated the correlation between expression levels of the miRNAs and clinical features. The results clearly demonstrated that miRNAs exhibited some changes in their expression levels along with cancer development and progression.

Our finding showed that, miR-21 is significantly upregulated in both plasma and matched tissue of CRC samples compared to healthy controls. Also, a ROC curve (AUC) of 0.98 for tissue miR-21 was found. Further analysis revealed that, the expression level of miR-21 was significantly different between an earlier stage (II) versus the late stages (stages III, IV). MiR-21, as an oncogenic miRNA (oncomiR) regulates a number of important indications of the tumor-progressing process.

Overexpression of miR-21 could enhance cell proliferation trough targeting PTEN and PI3K, whiledecreasing apoptosis via targeting BTG2, FasL and FBXO11. In addition, miR-21 has been shown to affect angiogenesis, metastasis, genetic instability and resistance to chemotherapy in several solid tumors including in CRC (19, 20). In agreement with our results, the expression level of this miRNA has been reported to be associated with clinical stage and survival of CRC patients (20-22). Accordingly, a potential role of miR-21 in tumor growth and progression of the malignancy, and as a diagnostic biomarker was confirmed more than ever.

We also found that the expression of miR-135b both in tumor tissue or plasma was significantly up-regulated in comparison with that of healthy controls, which was in agreement with earlier studies (13, 22). MiR-135b, as an oncogenic regulator in CRC modulates cell proliferation, apoptosis and chemoresistance through regulating key tumor suppressor genes such as LATS1, LATS2 and APC (22). A number of studies have witnessed a remarkable upregulation of miR-135b in CRC tissues (13, 22) of mouse or human tumors, suggesting it as a primary event in CRC (8).

Based on our results and pervious findings, miR-135b is believed to be involved in CRC development and progression (13, 22). However, tissue miR-135b with a specificity of 0.906 and a sensitivity of 0.973, and AUC of 0.91 showed a higher value than plasma miR-135b for cancer discrimination.

Nevertheless, one study from the Chinese population, provided no evidences that circulating cell-free miR135b could be considered as a biomarker for early CRC detection (23). These inconsistent results may be due to the different approaches in sampling. Recent studies suggested that plasma, but not serum, is the sample of choice in examining cell-free circulating miRNAs, because miRNAs might be released from blood cells into serum during the coagulation process changing the true spectrum of circulating tumor-derived miRNAs (17). In this study plasma samples were chosen as a more reliable source of extracellular cancer-related miRNAs.

In the present study, the upregulation of miR-31 in CRC tissue and plasma samples in comparison with healthy subjects was revealed. In addition, we showed a significant upregulation of miR-31 in all clinical stages of CRC compared to healthy controls, which is supported by other studies (13). Based on previous studies, miR-31 has been recognized as a potent cancer-related miRNA, involved in carcinogenesis of CRC by targeting tumor suppressor genes such as HIF-1a and CDKN2B (24, 25). This miRNA has also been demonstrated to act as an oncomiR in human CRC, where its overexpression is associated with cell proliferation, invasion and metastasis (13, 26). Our analysis, in line with the above- mentioned studies, showed that tissue miR-31 with a ROC AUC of 0.91 might serve as a potential diagnostic biomarker for CRC.

MiR-20a, as an upregulated miRNA in CRC, has been revealed to increase cell migration and metastasis by suppressing Smad4 and E-cadherin expression (27, 28). MiR-20a has also been shown to induce CRC cell proliferation trough suppression of the TGF-ß signaling pathway and inhibition of the G1/S checkpoint (28). Our results are consistent with the previous studies (27, 28) showing a significant overexpression of miR-20a in CRC plasma and tumor compared to the healthy group. We also observed tissue miR-20a with a ROC AUC of 0.92 had a stronger power than plasma miR-20a for cancer diagnosis. Altogether, there was a positive association between high plasma and tissue expression of miR-20a and advanced clinical stages of CRC, suggesting a probable oncogenic role for miR-20a in the pathogenesis of CRC.

miR-200c, as a well-known post-transcriptional regulator in many cancer cell signaling pathways, is involved in tumor proliferation, cell cycle control, invasion, and metastasis (29, 30). Also, it has been reported that miR-200c could act as an oncogene or tumor suppressor in different types of cancers, or even at different stages of a defined tumor (30). We observed a significant downregulation of miR-200c in CRC samples compared to the healthy group.

Our finding is not the first report of downregulation of miR-200c in CRC (31). Several functional studies have revealed that miR-200c inhibits epithelialmesenchymal transition (EMT) and cancer cell migration by downregulating ZEB1/2 and upregulating E-cadherins (31, 32). More recently, miR-200c has been shown to be downregulated at the invasive front of CRC while upregulated in the metastasis status (31). One rationalization for these findings may be the hyper- or hypomethylation trend of miR-200c through sequential steps of the tumor’s development (32). Notably, miR-200c expression levels were significantly lower in advanced stages (III and IV) than stage (II). These clinical findings supported the results from some related functional studies showing a downregulation of miR-200c in the initial stages of the disease (33).

Let-7g, has been reported to target major oncogenes as a potent suppressor. Oncogenes including RAS, HMGA2, BCL2L1 and GAB2 that affect cell proliferation and apoptosis pathways (34). The significant downregulation of this molecule has been observed in numerous cancers, including lung, liver, breast, gastric and colon cancer (34, 35). However, there are a few studies on the biological role of let-7g in the CRC (36). Our results exhibited significant reduced expression levels of let-7g in CRC compared to normal samples signifying this miRNA may act as a potential tumor-suppressor (37). Based on our findings and other investigations, this miRNA seems to be associated with clinical outcomes of colorectal cancer.

MiR-145 can function as a suppressor of cell proliferation and tumor metastasis through targeting multiple oncogenes such as MYC, Kras, IRS-1, SOX2, MUC1, etc. (38, 39), and its expression level has been shown in several cancer cell lines (40). A number of previous studies reported a significantly reduced level of miR-145 at the adenomatous and clinical stages of colorectal neoplasm (21, 26). 

We found a significant reduction in the expression of miR-145 in cancer patients in comparison with healthy controls. Based on these findings, this miRNA may be associated with clinical outcome of colorectal cancer. Taken together, our results indicated that the expression levels of miRNAs are systematically altered in CRC tissue and plasma samples. Moreover, the aberrant regulations of miRNAs are relatedto different clinical stages of CRC. The predictable changes of miRNA signatures may happen during tumorigenesis and may be representative of CRC development. These evidences are helpful in illuminating the molecular mechanisms underlying CRC carcinogenesis. On the other hand, the combination of the miRNAs-based biomarkers with other existing screening tests could be useful for diagnostic and prognostic accuracy as well as therapeutic planings. 

## Conclusion

Our results supported the hypothesis that plasma miRNAs expression pattern might reflect the expression pattern of their matched tissues. Our findings suggested that differential expression of miR-31, 20a and 135b and 21 may be serve as potential biomarkers for CRC detection.
